# Integrating operant behavior and fiber photometry with the open-source python library *Pyfiber*

**DOI:** 10.1038/s41598-023-43565-1

**Published:** 2023-10-02

**Authors:** Dana Conlisk, Matias Ceau, Jean-François Fiancette, Nanci Winke, Elise Darmagnac, Cyril Herry, Véronique Deroche-Gamonet

**Affiliations:** 1 University of Bordeaux, INSERM, Neurocentre Magendie, U1215, F-33000 Bordeaux, France; 2 UCL, Sainsbury Wellcome Centre, London, UK

**Keywords:** Fluorescence imaging, Optical imaging, Neural circuits, Addiction

## Abstract

Despite the popularity of fiber photometry (FP), its integration with operant behavior paradigms is progressing slowly. This can be attributed to the complex protocols in operant behavior – resulting in a combination of diverse non-predictable behavioral responses and scheduled events, thereby complicating data analysis. To overcome this, we developed *Pyfiber*, an open-source python library which facilitates the merge of FP with operant behavior by relating changes in fluorescent signals within a neuronal population to behavioral responses and events. *Pyfiber* helps to 1. Extract events and responses that occur in operant behavior, 2. Extract and process the FP signals, 3. Select events of interest and align them to the corresponding FP signals, 4. Apply appropriate signal normalization and analysis according to the type of events, 5. Run analysis on multiple individuals and sessions, 6. Collect results in an easily readable format. *Pyfiber* is suitable for use with many different fluorescent sensors and operant behavior protocols. It was developed using Doric lenses FP systems and Imetronic behavioral systems, but it possesses the capability to process data from alternative systems. This work sets a solid foundation for analyzing the relationship between different dimensions of complex behavioral paradigms with fluorescent signals from brain regions of interest.

## Introduction

There has been remarkable progress in the last twenty years regarding tools that monitor activity in specific neuronal populations using fluorescent indicators, especially applied to rodent models. The emergence and continuous improvement of genetically encoded calcium indicators (GECIs), specifically GCaMPs, have allowed monitoring specific neuronal populations in real-time^1^. By binding to calcium ions and emitting fluorescence that visualizes intracellular calcium concentration, GECIs enable detection of in vivo neuronal firing activity.

In parallel, recent developments in genetically encoded biosensors permit neural activity dissection by observing various markers^2^. In addition to GECIs, genetically encoded voltage sensors, pH sensors, and neurotransmitter indicators, such as dLight for dopamine, have been developed^3^. This, paired with expertise in viral genetics, has permitted selective expression of these GECIs in subtypes of neuronal populations allowing researchers to observe alterations of specific neuronal subtype’s activity in vivo^4,5^.

The use of these fluorescent indicators with bulk imaging techniques such as fiber photometry can be highly beneficial in identifying neuronal signatures in behavioral neuroscience experiments and explains its rapidly growing popularity. However, the application of fiber photometry to operant behavior is still limited. Technical constraints, such as freely-moving behavior while head-connected, are now relatively minor difficulties, evidenced by the expansion of coupling of operant behavior with in vivo optogenetics. For neural imaging, the main challenge for behavioral neuroscientists is to optimize data analysis by dealing with analytical constraints. In operant behavior, unpredictable behavioral responses add difficulty in analysis due to their unsystematic distribution. Due to this complexity, the analyses tend to be centered on events or behavioral responses with a highly-controlled trigger, restricting the results.

Extraction of information from fiber photometry recordings during operant tasks relies on the ability to successfully apply the following steps:

1. Extract the different types of scheduled and unscheduled events that occur in the operant session,
2. Extract and process the fiber photometry signals,
3. Select all events or behavioral responses of a given type either globally or within a given time interval of the operant session,
4. Align events of interest to corresponding fiber photometry signals,
5. Apply the most appropriate type of signal normalization and signal analysis to study the neuronal response associated with a given type of event or behavioral response,
6. Run data extraction and analysis on multiple sessions at the same time,
7. Collect results in an easily readable format for statistical analysis.

In this objective, we created *Pyfiber*, a Python library that was originally created to extract and relate the data from our operant behavior (Imetronic polymodal operant boxes) and fiber photometry (Doric Lenses Inc.) systems. However, we have expanded it to allow importation of other behavioral and fiber photometry systems.

*Pyfiber* can be integrated into a homemade command-line application and interfaced in a notebook. We recommend using the Anaconda environment and more specifically, Jupyter notebook (see details in the “Methods”).

## Materials and methods

### Ethics approval

All procedures involving animal experimentation and experimental protocols were approved by the Animal Care Committee of Bordeaux (CEEA50, #18135) and were conducted in accordance with the guidelines of the European Union Directive 2010/63/EU regulating animal research.

### General experimental procedures

#### Cocaine self-administration

**Figure 1:**
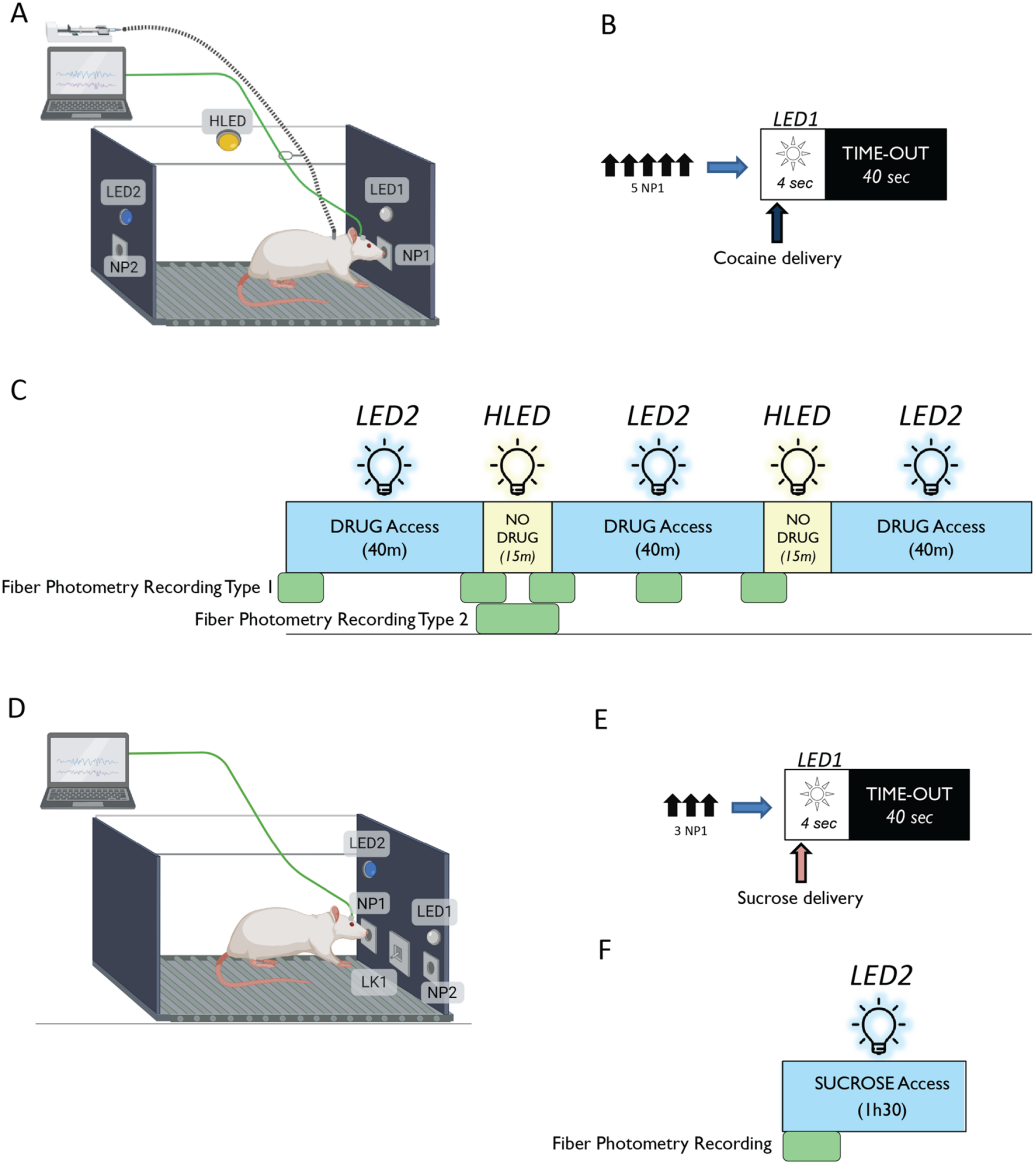
Schematic of the operant set-up and protocols. (**A**) Cocaine self-administration: Schema of a rat within a self-administration box featuring operant manipulanda (Holes NP1 & NP2) and three distinct lights (LED1, LED2 & HLED). The rat’s intravenous catheter is connected to a pump-driven syringe via a flexible spring (dashed dark line). The box is equipped with holes used as operant manipulanda and with lights used as stimuli. The implanted optic fiber is connected to the Doric recording system through an optical fiber patch cord (green line). (**B**) Responding is based on a Fixed Ratio 5 (FR5) schedule, which requires the rat to nose-poke into the active hole (NP1) five times to receive an injection. One second after the fifth nose-poke, the conditioned stimulus (LED1) illuminates for 4 s. One second after the onset of illumination, the pump is activated, delivering cocaine intravenously over 2 s. A subsequent time-out period of 40 s ensues, during which no lights are illuminated. Nose-pokes in the opposite hole (NP2) do not yield any scheduled consequences. (**C**) Daily sessions consist of three 40-min drug periods (indicated by the illumination of LED2) (Drug DS) separated by two 15-min drug-free periods (indicated by the illumination of HLED) (No Drug DS). The green blocks beneath the self-administration diagram denote fiber photometry recordings. (**D**) Sucrose oral self-administration: Schema of a rat in the self-administration box equipped for sucrose self-administration. Conditions are similar to cocaine self-administration; however the sucrose solution is dispensed to a licker within the cage. (**E**) Following three nose-pokes in the active hole, the conditioned stimulus (LED1) illuminates for 4 s. One second after the onset of illumination, the pump is activated, delivering sucrose to the licker. A subsequent time-out period of 40 s ensues, during which no lights are illuminated. Nose-pokes in the opposite hole (NP2) do not yield any scheduled consequences. (**F**) Daily sessions consist of a 90-min sucrose access period (indicated by the illumination of LED2). Fiber photometry recording takes place during the first 15 min of a session. Created with BioRender.com.

The context of our research is the vulnerability to develop cocaine addiction and its neurobiological underpinnings. We use a preclinical model of addiction based on operant intravenous self-administration behavior in rats^6^. Rats are equipped with an indwelling catheter in the jugular vein and placed daily in an operant box where their catheter is connected to a pump-driven syringe. Upon nose-poking into an active hole (NP1) (Fig. 1A) according to a specific schedule (Fixed ratio 5, time-out 40 s) (Fig. 1B), rats receive intravenous cocaine injections during three 40 min signaled periods (drug periods) alternating with two signaled periods of drug unavailability (no drug periods) (Fig. 1C). Fixed Ratio 5 of responding (FR5) means that the rat needs to nose-poke in the active hole (NP1) 5 times to receive an injection. To prevent possible overdoses, a time-out period of 40 s occurs after each injection, during which active nose-pokes are recorded but have no consequences. Nose-poking in the inactive hole (NP2) is without scheduled consequences at any time. We couple this operant behavior with GCaMP-based calcium imaging through FP (Fig. 1A,C).

#### Sucrose self-administration

Additionally, our research uses sucrose self-administration protocols. Rats are placed in an operant box for daily 90 min sucrose-access sessions (Fig. 1D). Upon nose-poking in an active hole according to a specific schedule (Fixed ratio 3, time-out 40 s) (Fig. 1E), a 10% sucrose solution is delivered to the licker in the operant cage. We couple this operant behavior with dLight-based fluorescent imaging through FP (Fig. 1D and F).

Subjects, Surgeries, Histology, Self-administration (SA) apparatus, Self-administration training, Fiber photometry system and sensors, Fiber recording during self-administration are described in the Supplementary Information (SI).

### Behavioral data processing (Imetronic)

Pyfiber processes raw data files natively from Imetronic systems. Behavioral data is outputted by Imetronic as a tab-separated values text file, where each row represents a different message encoded by integers in each column. The first step consists of extracting and translating the data. The information retrieved is timestamps for the different experimenter-scheduled or animal-produced events related to the defined operant protocol [(ex. of scheduled event: hled_on = moment the house light turns on signaling shift from drug to no drug period; ex. of animal-produced events: nose-pokes in active and inactive holes, injections, injection-associated conditioned cue light stimulus (CS)], as well as experimenter-scheduled intervals (ex. of interval: HLED_ON = the time period in which the house light is on).

From this raw data, specific custom experimenter-defined events and intervals are computed to be used for analysis. First, intervals corresponding to the different periods of time are algorithmically retrieved from the combination of light activation, and event/time-based rules. This strategy is particularly useful for intervals occurring at unpredictable times, such as time-outs following a drug injection. In addition, it also allows the accurate determination of timestamps for experimenter-defined periods (such as the fixed drug and no drug periods), which is crucial for later alignment with fiber photometry data. Finally, a variety of more specific information (such as the position of each nose-poke in the fixed ratio series, or the first nose-poke in a given interval) can be retrieved. All of these events are defined and can be modified in the configuration file of *Pyfiber* (see Results-Configuration File and 2.2 in the SI).

Both the general extraction of events and intervals, as well as custom experimenter-defined events and intervals exist due to some behavioral protocols have more complex events occurring. For example, in our behavioral protocol, the house light (HLED), when on, indicates the no drug period (Fig. 1C). Normally, the switch from the drug to no drug period happens on the switch from the drug period light (LED2) to the no drug period light (HLED), however if the animal happens to be in a post-injection time-out period (Fig. 1B), the switch from the drug to the no drug period will be from darkness to the no drug period (darkness to HLED), which should not be compared to recordings in which the switch is occurring from the drug period (LED2) to the no drug period (HLED). Due to this, we added custom criteria within the configuration file to specifically identify these two different types of light-switch occurrences.

### Behavioral data processing (other behavioral systems)

Pyfiber allows the analysis of data from systems other than Imetronic. These files must adhere to a specific structure, where events are organized in columns and timestamps, or specific values related to non-temporal events, are arranged in rows in CSV format. This format is outlined in Table 1. The headers correspond to the names of the events while the rows contain the timestamps of the events.

**Table 1:** Example data format that behavioral data must adhere to in order to be integrated into *Pyfiber*.

Dim1_on	,	Dim1_off	,	Dim2
timestamp#1	,	timestamp#1	,	timestamp#1
timestamp#2	,	timestamp#2	,	timestamp#2
…	,	…	,	…

A complete example of the necessary structure, based on the data from the bsaRat12_28032022c20_01.dat file, can be found in the git repository.

It is important to note that despite the data being organized in a CSV format, the file extension should remain as .dat due to technical constraints. In addition, unlike Imetronic data, only basic and custom intervals should be configured in the .yaml configuration file. Event names are directly extracted from column headers of the data file. These names must be unique and follow standard ASCII characters (refer to Section 2.2 SI).

### Fiber photometry data processing

#### Input files

The files issued from the FP Doric system can be .csv or .doric (.HDF5) depending on the version of *Doric Neuroscience Studio* (exclusively .doric starting version 6). Pyfiber can manage both types of files. If other fiber photometry systems are used, they need to be formatted in the same way as the doric .csv.

#### Signal processing

The exact methodology of normalization of the calcium-dependent and isosbestic (i.e. control) channels differs between labs, therefore we implemented multiple ways of signal processing into our library.

##### ΔF/F

The current standard in the literature is based on a linear regression model^7,8^. The assumption is that outside of the events that trigger depolarization of neurons, and thus calcium release, the signal and control channels will be strongly correlated. Thus, in order to scale the control to the signal, a linear regression is performed on with data from the two channels with the least square method^8^. The regression coefficients are then used to scale the isosbestic channel to the calcium-dependent channel (i.e. signal) by using Eq. (1). The resulting value corresponds to an activity ratio compared to baseline, although different factors such as random noise and calcium indicator decay may render it less straightforward.

1 $$\frac{\Delta F}{{F}_{0}}= \frac{signal-fitted \,\,isosbestic}{fitted\,\, isosbestic}.$$

##### Z-scores

The second method consists of calculating Z-scores for the two signals, which can then be subtracted to obtain a unique normalized signal (Eq. 2).

2 $$Z=\frac{signal-mean\,\, signal}{SD\,\, signal}-\frac{isosbestic-mean\,\, isosbestic}{SD\,\, isosbestic}.$$

#### Peak analysis

In addition to the magnitude of the calcium-signal (reflecting a global increase in activation in the region of interest), the frequency of transients can also provide useful information. A transient is defined as a sudden increase in signal, which reflects neural activity. However, due to random noise, artifacts, and other constraints of fiber photometry, the normalized signal contains many local maxima, many of which are irrelevant. Thus, a strategy to determine if a transient is a true peak (signifying depolarization) was implemented. Many different methods exist in the literature but *Pyfiber* implements the most commonly used^9,10^. The signal is analyzed with a default of 10 s windows to limit the impact of signal decay caused by photo-bleaching, which would lead to an underestimation of peaks towards the end of the recording. Standard scores are either calculated for the whole recording or inside each time window. Then, inside each window, the baseline median absolute deviation (MAD, or bMAD) is calculated, and a first threshold is set (most commonly 2 to 2.5 times the MAD, default of *Pyfiber* = 2.5). Signal data below this first threshold is removed, and a new threshold is based on the MAD of the remaining data (pMAD, most commonly equal to 3.5 times this second MAD, default of *Pyfiber* = 3.5). All signal data higher than this second threshold are considered as transients. However, as the sampling rate of the fiber photometry data (in our case around 1000 Hz) is faster than the rise time of the GECI we use (GCaMP6f- 50 ms, or 20 Hz), the transients needs to be filtered to extract only the peaks from single events. Therefore, only the highest value from a 50 ms window is extracted and considered as a peak.

#### Perievent analysis

A major aim of the combination of operant behavior and fiber photometry is to determine whether the neural activity before or after a particular event (for example a light switch or a nose poke triggering a cocaine injection) is significantly different than at baseline, as well as comparing between subjects. In order to evaluate this, additional data processing surrounding events (perievent analysis) is performed.

##### Z-scores

To do so, Z-scores (Eq. 3) are calculated by taking the time preceding the event as the baseline. Depending on which method was initially used for signal processing, perievent Z-scores can be generated from either the ΔF/F or Z-score.

3 $${Z}_{peri}=\frac{{\frac{\Delta F}{{F}_{0}}}_{perievent}- mean ({\frac{\Delta F}{{F}_{0}}}_{baseline}) }{SD ({\frac{\Delta F}{{F}_{0}}}_{baseline})}.$$

##### Robust Z-scores

For dispersed data, outliers can have a detrimental effect on both the mean and the standard deviation. Thus, another method has been proposed^7^. It utilizes robust Z-scores (Eq. 4), which are similar to classical Z-scores but use the median instead of the mean and the median absolute deviation (Eq. 5) instead of the standard deviation. This has the effect of removing the influence of outliers on the analysis.

4 $${Robust \,\,Z}_{peri}=\frac{{\frac{\Delta F}{{F}_{0}}}_{perievent}- median \left({\frac{\Delta F}{{F}_{0}}}_{baseline}\right)}{MAD ({\frac{\Delta F}{{F}_{0}}}_{baseline})},$$

5 $$MAD = median(\left|Xi - median\left(X\right)\right|.$$

## Results

### Summary of *Pyfiber*

**Figure 2:**
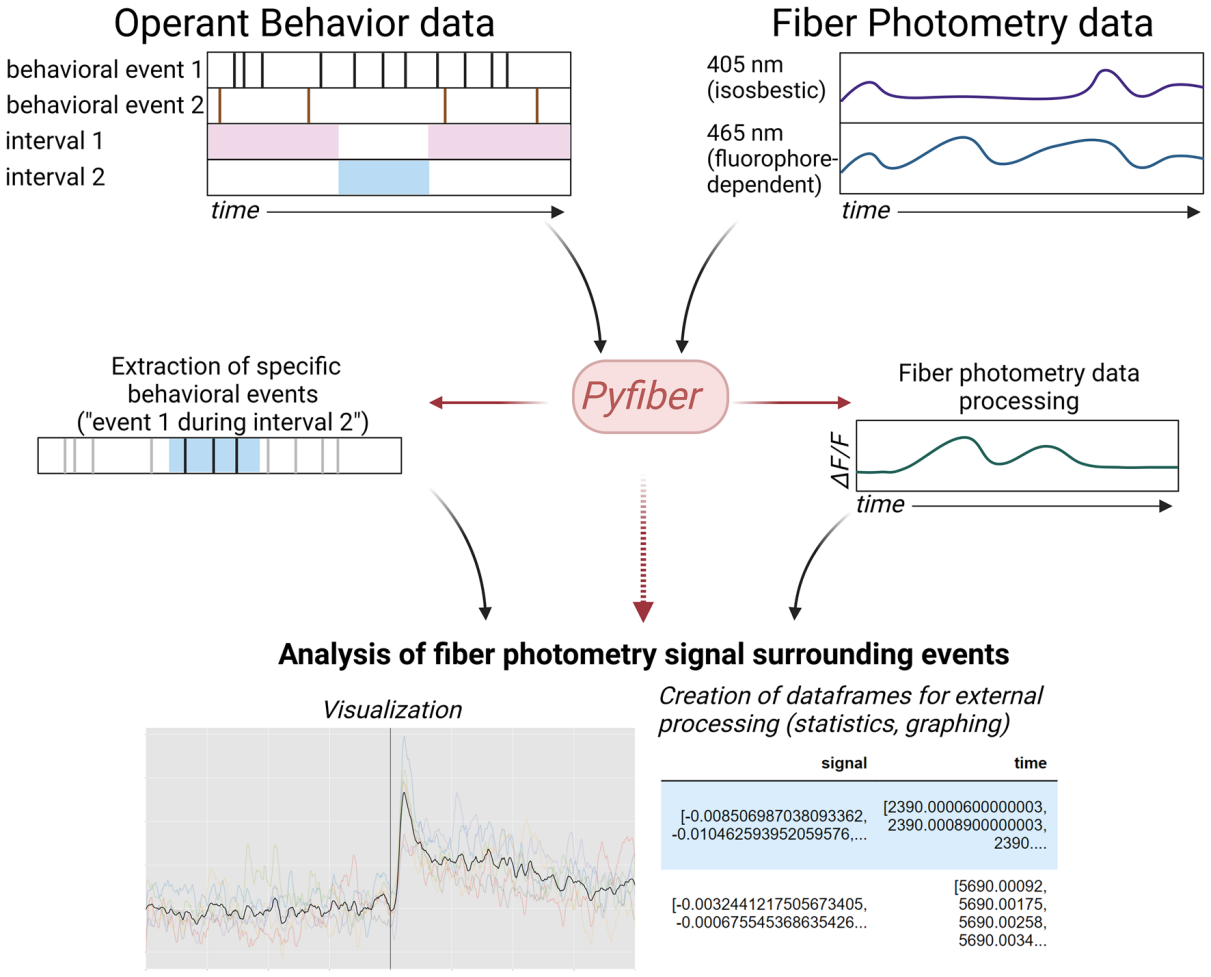
Schematic of *Pyfiber* operating mode. *Pyfiber* requires operant behavior file(s) and fiber photometry data file(s). From these files, *Pyfiber* can extract specific behavioral events and process the raw fiber photometry data. The main practicality of *Pyfiber* is the peri-event analysis of the fiber photometry signal surrounding events of interest that were extracted from the raw behavioral data. Created with BioRender.com.

*Pyfiber* was created to fill the gap in flexible, open source analytical tools merging operant behavior and fiber photometry (Fig. 2). *Pyfiber* requires inputs of behavioral files (Imetronic or formatted data from other behavioral systems) and fiber photometry files (Doric Lenses, .csv or .doric). *Pyfiber* dissects and stores the data within the behavioral file by identifying and categorizing events and intervals. Additionally, *Pyfiber* dissects and stores the data in the photometry file by processing the photometry signals using previously established and reputable photometry analysis methods. Then, it uses the aforementioned stored data (both behavior and fiber photometry) to fuse the stored data, resulting in the output of processed fiber photometry data surrounding the events of interest that were extracted from the behavioral file. The merge of the behavior and fiber photometry data results in a multitude of possibilities, including but not limited to visualization of the data, extraction of dataframes containing the individual and average signals [either the pre-processed signal (ΔF/F) or re-normalized peri-event signal (Z-scores or Robust Z-scores)].

#### Operating mode: introduction to *Pyfiber* configuration file and operational modules

##### The configuration file

[in .yaml format (https://en.wikipedia.org/wiki/YAML)] describes the different phases and events of a behavioral session. If an event of interest is not already implemented in the configuration file (for example, a lever press), it can be defined and added by inserting the Imetronic nomenclature for the event.

If non-Imetronic data files are being utilized, events are identified by specifying their names (corresponding to the header in the behavioral data file containing the timestamps) in the 'csv_events' section within the behavioral section.

It also describes the structure of the data from the fiber photometry system, as well as all the events of interest used in the analyses. It also contains the default analysis settings. Therefore, changing the configuration file will alter the default *Pyfiber* processes. Video 3 shows how to modify the configuration file if needed. For a more detailed description of the configuration file, see the supplementary information.

##### The three operational modules

*The* *analyze* *module*: The Analyze module analyzes the combination of behavioral data and Doric Lenses fiber photometry data. It extracts identical behavioral responses or events occurring during fiber recordings and performs fiber perievent analysis on them. However, it is not used independently- it is always called by *Pyfiber* when either the MultiSession or Session modules are called.

Session module: The session module imports a single session [one fiber data file and one behavioral file] for analysis of a single timestamp. The Session module allows for perievent analysis of an indicated timestamp that can be extracted from the behavioral file. Therefore, if the interest is looking at a single event in a single session, the Session module is the most useful.

MultiSession module: The MultiSession module imports a single or multiple sessions (each session as a folder containing one fiber data file (.csv or .doric) and its associated behavioral data file (either a raw Imetronic .dat file or a formatted data file from other systems) to perform group analysis. This can also be done with a single session that contains multiple events of the same type (for example, one session containing multiple injections). This permits simultaneous analysis of multiple recordings and events quickly and easily. The results can be exported to be graphed or for statistical analysis. This is the most complex function of *Pyfiber* and the most commonly used in our lab. For example, suppose the goal of the analysis is to see transients in response to a light presentation signaling cocaine delivery. In that case, MultiSession will be able to extract the time of every light presentation from the behavioral files and refer to each associated fiber photometry recording to perform perievent analysis.

*The* *fiber* *module*: The fiber module is used by the Analyze modules (both MultiSession and Session). This module is responsible for processing the fiber photometry data in preparation for perievent analysis. However, suppose there is a specific interest in observing the fiber photometry data independently. In that case, the Fiber module can be useful and can be used to analyze a single fiber photometry recording file.

*The* *behavior* *module*: The behavior module is also used by the Analyze module. It either extracts events and their timestamps from the raw Imetronic data file or loads the events and their timestamps from specifically formatted behavioral data files from other systems. This is done in preparation for perievent analysis by the Analyze module(s). Like the Fiber module, the Behavior module can be used independently if there is a specific desire to look solely at behavioral events. It can be used in the two different following modes:

Behavior module: The behavior module is used to look at a single data file. This is used in both the MultiSession and Session modules to extract timestamps for the associated perievent analysis. It can be used independently to extract behavioral responses or events from a single operant session.

MultiBehavior module: The MultiBehavior module is used to compare behaviors between subjects, groups or sessions using multiple data files. The MultiSession module can be used to visualize how the behavioral activities differ between the sessions that will be analyzed for perievent fiber photometry analysis.

#### How to install *Pyfiber* on your computer

Video 1 shows how to install Anaconda, Jupyter notebook and *Pyfiber* on your computer, step by step (see also SI for more help). A repository hosting useful links on how to install and use Pyfiber, as well as example data *(Gitlab)* can be found here: https://gitlab.com/inserm-u1215/pyfiber.

#### How to run analyses with *Pyfiber*

As stated above, the MultiSession module is the prototypical use of *Pyfiber* when running fiber photometry recordings during an operant task over several sessions and/or on several individuals. It analyzes multiple behavioral and fiber data files in parallel. It uses the **Analyze,** the **Behavior** and the **Fiber** modules. For the sake of space, only tutorials on the MultiSession Analyze module are described below. For tutorials on Fiber and Behavior (MultiBehavior, Behavior) modules, and the Session Analyze Module, please see the Jupyter Notebooks associated with those modules, and the SI. Tables in the SI contain a selection of the most pertinent features and the commands associated with the different analyze modules.

An example is given on 3 recordings run during 3 independent behavioral sessions (all run on the same rat). The corresponding Jupyter Notebook can be found and has to be downloaded together with the .csv (fiber data) and .dat (behavior data) files at: https://gitlab.com/inserm-u1215/pyfiber/-/tree/main/notebooks/MultiSession.

Two prototypical types of analyses are detailed step by step in Video 2. The first analyzes an experimenter-scheduled event (shift from no drug to drug period signaled by the shift between two distinct light conditions (LED2 to HLED, see Fig. 1). In contrast, the second presents the analysis of non-scheduled events resulting from the animals’ behavior (in the present case: nose-pokes in the active hole or NP1 during drug periods).

The following steps shown are the only ones necessary for these examples but are excerpts of a Jupyter Notebook containing a variety of analyses. The corresponding locations of the steps below in the MultiSession Jupyter Notebook are indicated in the comment just above the execution of code.

*Step 1* (MultiSession Jupyter Notebook: #1): Import *Pyfiber* as following:
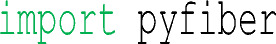


*Step 2* (MultiSession Jupyter Notebook: #2): Create a MultiSession object (here named ‘ImportedFiles’) by calling pyfiber.MultiSession and indicating the path to the folder containing the subfolders with the behavioral and photometry files within parenthese as following:
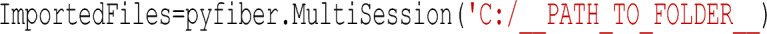


When working with systems other than Imetronic, a second parameter should be passed as the 'filetype' parameter:
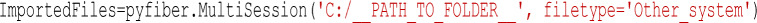


*Step 3* (MultiSession Jupyter Notebook: #8): Create a perievent object by calling .analyze from the MultiSession object. Indicate in the parenthesis which events (scheduled or non-scheduled) should be used as the events of interest, the interval of interest if relevant, the window that should be used surrounding the event, and the normalization type.

i. An experimenter-scheduled event.

In this section, we are interested in the shifts from drug to no drug period **(**signaled by HLED illumination**)**. The scheduled events are therefore determined by ‘hled_on’, our window of interest is from − 5 s to + 5 s centered on the event. The normalization type is the standard DeltaF/F called ‘F’. The perievent object is named here, as an example, as HLED5_5 and coded as following:
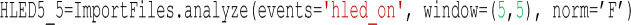

ii. A non-scheduled event produced in a given period of the behavioral session.

Here we analyze whether the nose-pokes produced by rats in the active hole (np1), during the drug periods, generate a specific FP signal.

**Figure 3:**
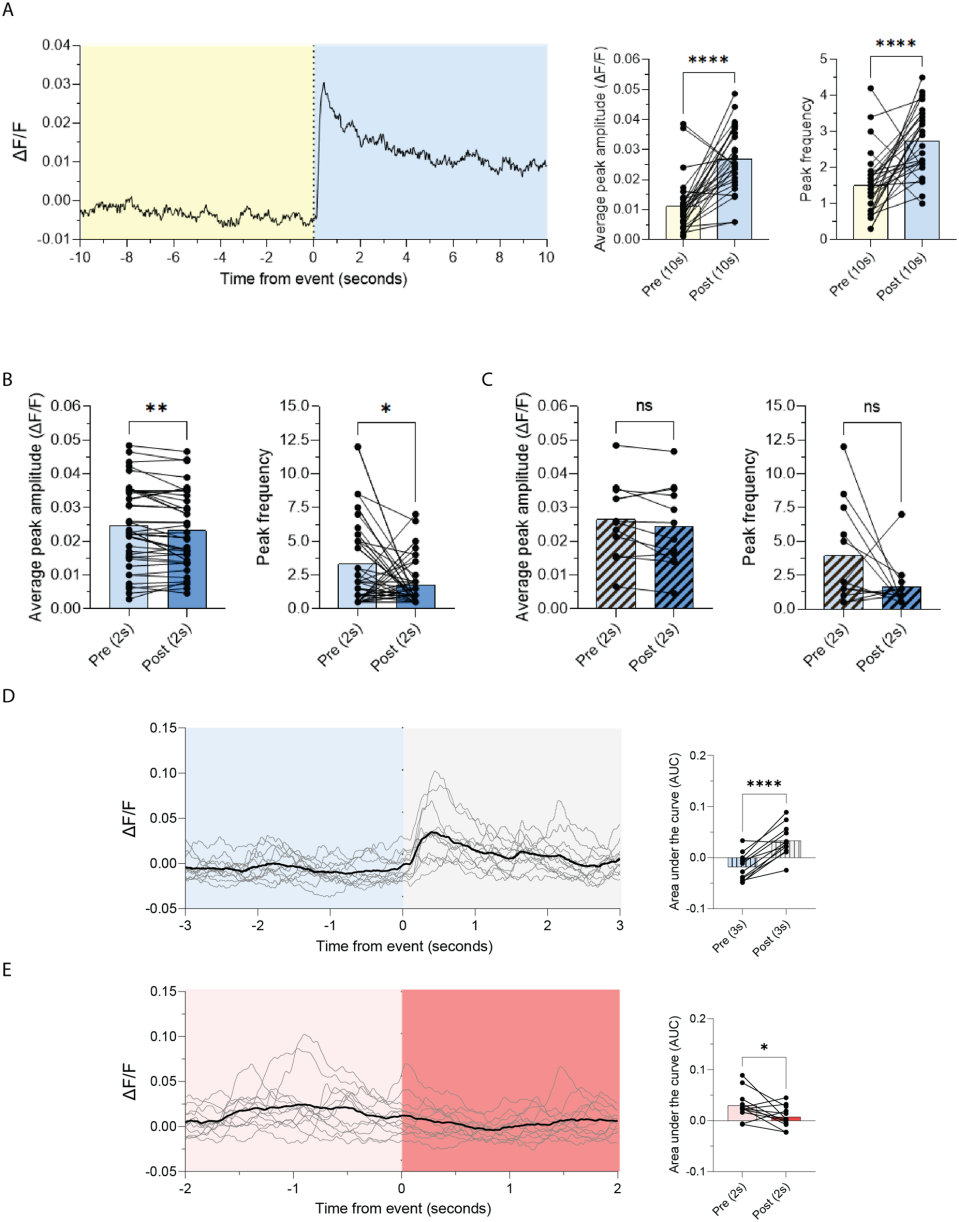
Examples of results obtained through the use of *Pyfiber* (**A**) The average trace of the GCaMP6f signal during the shift from the No drug period to the Drug period (10 s pre/post) (left). Average peak amplitude (ΔF/F) and peak frequency analysis are also presented for the same shift (right). (**B**) Average peak amplitude (ΔF/F) and peak frequency analysis are presented for the changes in calcium transients associated with all active nose-pokes (NP1) performed during the second Drug period recording (2 s pre/post). (**C**) Average peak amplitude (ΔF/F) and peak frequency analysis are presented for the changes in calcium transients associated with the first nose-pokes of the FR5 series during the second Drug period recording (2 s pre/post). (**D**) The average trace of the dLight1.3b signal associated with the cue presentation produced when the rat reached FR3 during a sucrose self-administration operant paradigm (left) and the pre/post area under the curve (AUC) for the events (3 s pre/post) (right). (**E**) The average trace of the dLight1.3b signal associated with the first lick that followed each sucrose delivery (left) and the pre/post area under the curve (AUC) for the events (2 s pre/post) (right).

The events correspond to ‘np1’, our intervals of interest are the drug periods corresponding to the periods of time where the light LED2 is on, our window of interest is from − 1 s to + 1 s centered on the event. The normalization type is the standard DeltaF/F called ‘F’. The perievent object is named here, as an example, as NosepokeDuringDrugPeriod, and coded as following:
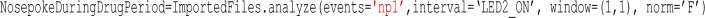


*Step 4* (MultiSession Jupyter Notebook: #9): To plot the perievent results, call .plot from the perievent object and indicate what to plot to see the individual (colored) and average (black) traces of the perievent signal. We use here as examples the data shown in Fig. 3. Figure 3A(left) shows the averaged plot for a scheduled event (shift from No drug to Drug period, “led2_on”). In Fig. 3D and E(left) are shown individual and averaged plots for non-scheduled events (Cue presentation (“led1_on”) and the first lick (“lk1”) that followed each sucrose delivery, respectively).

*Step 5* (MultiSession Jupyter Notebook: #10): to see a table containing the average values for the perievent signal: including pre/post area under the curve (AUC), dF/F, Z-Scores, Robust Z-Scores, peak frequency, peak amplitude, etc…, call .data from the perievent object. For the complete list of things that are in this table, see .data in the MultiSession table found in the SI (Table 2 shown for the scheduled event). Example paper-ready figures that can be extracted and plotted from this data table are shown in Fig. 3. Figure 3A(left) and B,C show perievent analysis on the average peak amplitude and frequency of both scheduled/unscheduled events in cocaine self-administration, while Fig. 3D,E (right) show perievent analysis on the area under the curve (AUC) of both scheduled/unscheduled events in sucrose self-administration.

**Table 2:** Example excerpt from the dataframe obtained from *Pyfiber* when calling HLED5_5.data in Step 5 of the example analysis.

	Behavior file	Event_time	Fiber file	Normalization	postAUC	PostAVG_RZ	PostAVG_Z	postAVG_dF
AS21R_rat_12_SA5_j32	C:/Users/Optopath2/Desktop/notebooks/ …	2400.000	C:/Users/Optopath2/Desktop/notebooks/ …	F	0.081542	2.035268	1.440327	0.016305
AS21R_rat_12_SA5_j32	C:/Users/Optopath2/Desktop/notebooks/ …	5700.001	C:/Users/Optopath2/Desktop/notebooks/ …	F	0.170655	4.760854	3.180732	0.034127
AS21R_rat_12_SA6_j36	C:/Users/Optopath2/Desktop/notebooks/ …	2400.001	C:/Users/Optopath2/Desktop/notebooks/ …	F	0.150752	6.817647	4.873567	0.030145
AS21R_rat_12_SA6_j36	C:/Users/Optopath2/Desktop/notebooks/ …	5700.004	C:/Users/Optopath2/Desktop/notebooks/ …	F	0.089828	4.611997	3.047546	0.017964
AS21R_rat_12_SA7_j40	C:/Users/Optopath2/Desktop/notebooks/ …	2400.008	C:/Users/Optopath2/Desktop/notebooks/ …	F	0.112181	4.934199	3.098110	0.022433
AS21R_rat_12_SA7_j40	C:/Users/Optopath2/Desktop/notebooks/ …	5700.018	C:/Users/Optopath2/Desktop/notebooks/ …	F	0.121177	2.813025	1.750664	0.024229

This table contains the average values for the perievent signal including, but not limited to pre/post area under the curve (AUC), dF/F, Z-Scores, Robust Z-Scores, peak frequency, and peak amplitude.

*Step 6* (MultiSession Jupyter Notebook: #12): to see a table that contains the dataframes for the perievent analysis, call .full_data from the perievent object (Table 3 shown for the scheduled event).

**Table 3:** Example excerpt from the dataframe obtained from *Pyfiber* when calling HLED5_5.full_data in Step 6 of the example analysis.

raw_control	raw_signal	Raw data	rec_number	recordingdata	rob_zscores	sampling_rate
[0.140690329, 0.140690329, 0.140690329, …]	[0.154728843, 0.154423658, 0.154423658, …]	[[2281.0212, 0.179754021, 0.160832545], [2281…	2	[[2281.0212, 0.00109965], [2281.0220…	[-0.4298407696, -0.691597788, …]	1212.489694
[0.133060701, 0.132755516, 0.132755516, …]	[0.146794031, 0.146794031, 0.147099216, …]	[[5581.05255, 0.169377728, 0.152897732], [5581…	5	[[5581.05255, -0.01453849], [5581.05…	[-1.7473492, -1.26824196, …]	1212.494544
[0.146488845, 0.146488845, 0.146488845, …]	[0.187994018, 0.187994018, 0.187994018, …]	[[2281.0212, 0.179754021, 0.160832545], [2281…	2	[[2281.0233, 0.007194596], [2281.0241…	[2.4745001, 2.4745001,, …]	1212.489694

This table can be of particular interest because it contains dataframes containing the raw and processed signal.

*Step 7* (MultiSession Jupyter Notebook: #15): Lastly, an example of the extraction of the dataframes containing the adjusted timestamps leading up to the event and the processed signal that corresponds to the timestamps (Table 4 shown for the scheduled event). These can be exported to a .csv file so the data can either be used in statistical programs or to create figures (see step 8).

**Table 4:** Example excerpt from the arrays obtained from *Pyfiber* when calling HLED5_5.interpolated_epoch and HLED5_5.interpolated_signal in Step 7 of the example analysis.

[array([– 9.9994, – 9.99917526, – 9.99835052, … 10.]), array([– 10., – 9.99917526, – 9.99835052, … 10.]), array([– 9.9994, – 9.99917526, – 9.99835052, … 10.])..]
[array([– 0.00850699, – 0.01030884, – 0.00879817, … 0.02800474.]), array([…])…]

This table is shown as *Pyfiber* displays it.

Since the tables can be quite large, some columns and rows might not be shown with the default display settings. The display settings can be changed using pandas, the data analysis module for the Python programming language.

Pandas is included in Pyfiber and the method to change display settings using pandas is shown in Video 2.

*Step 8* How to extract arrays: All of the data can be exported in multiple file formats depending on the user's needs. This can be achieved by using Python’s built-in functions or dedicated libraries such as pandas. The different steps to export the data to a .csv are detailed in Video 2.

#### Relevant information for all modules

In all modules, there is guidance available by calling < obj > .help or < obj > .info.

#### Example data obtained using *Pyfiber*

In Fig. 3, we show example data analysis using GCaMP6f in the prelimbic cortex paired with intravenous cocaine self-administration paradigm (Fig. 3A–C) as well as example data analysis using dLight1.3b in the nucleus accumbens paired with oral sucrose self-administration (Fig. 3D,E). Many different types of analysis can be extracted, from traces of the signal (Fig. 3A,D,E left) to quantification of peak frequency/amplitude (Fig. 3Aright, B,C) to the area under the curve (AUC) (Fig. 3D,E right).

Regarding the GCaMP6f sensor in the prelimbic cortex, the photometry recordings took place at different times (see Fig. 1) during 2h30 cocaine intravenous self-administration sessions alternating three drug periods (40 min) and two no drug periods (15 min). 29 recordings from 14 rats are featured on Fig. 3. Average peak amplitude (ΔF/F) and peak frequency analysis are presented for the response to the shift from No drug period 1 to Drug period 2 (Fig. 3A right). Average peak amplitude (ΔF/F) and peak frequency analysis are also presented for the changes in calcium transients associated with all active nose-pokes (NP1) performed during the second Drug period recording (Fig. 3B), and then specifically focusing on the first nose-pokes of the FR5 series (Fig. 3C).

Regarding sucrose oral self-administration, 2 recordings in 1 rat are analyzed. Figure 3D(left) shows the changes in dopamine transients associated with the cue presentations elicited when the rat reached FR3. Figure 3E(left) shows the changes in dopamine transients associated with the first lick that follow each sucrose delivery. In each case, the effect is evaluated through the comparison of the AUC (Fig. 3D,Eright).

## Discussion

Many papers have now started to show the benefits of fiber photometry paired with certain aspects of operant behavior^11–13^. The research pairing photometry with operant behavior is still in its infancy but has immense potential due to the diversity of events occurring during operant behavior- but this also serves as a barrier that complicates data analysis. However, with appropriate analytic efficiency, the combination of techniques can uncover neural signatures surrounding drug/reward administration, lever pressing/nose-pokes, and conditioned/discriminative stimuli presentation/changes. However, this relies on proper identification and tagging of behavioral events, which can be complicated when using complex behavioral paradigms. This led us to create *Pyfiber*, a python library for joint behavioral and fiber photometry analysis with the idea of complex operant behavioral paradigms in mind (such as- but not limited to- intravenous self-administration).

### Current open-source tools for joint behavioral/fiber photometry analysis

While open-source tools exist that aim to analyze fiber photometry recordings surrounding behavioral events, there were significant limitations that prevented us from using them. The pMAT program, despite having a very user-friendly interface, was quite rigid regarding the types of analysis performed^7^. For example, signal processing was limited to ΔF/F, without the possibility of other techniques. A main limitation of pMAT is that despite the .exe being executable without Matlab, modification of the pMAT program are not truly open-source due to the requirement to have a Matlab license.

GuPPY, another open-source fiber photometry tool, remedied a few of the aforementioned issues that were encountered with pMAT^14^. GuPPY was created in python, making it a truly free and open-source tool for fiber photometry analysis. Additionally, the tool implements many more options for analysis, such as transient frequency/amplitude analysis and other ways to process the raw fiber photometry signals. However, with GuPPY, the timestamps need to be extracted before analysis occurs, which can be a complicated step when using complex operant behavior paradigms.

### Justification of the creation of *Pyfiber* for joint behavioral/fiber photometry analysis

Various open-source packages providing toolkits for fiber photometry analysis exist, and the aforementioned programs have been made to provide easy-to-use software. However, specialized toolkits, often developed for task-specific applications, often lack generalizability, whereas more user-friendly projects appear to be more limited in their possibilities. As a growing field, fiber photometry still lacks a specialized code library that could provide the relevant tools for any type of fiber photometry data analysis projects. The modular approach of this project helps to achieve this aim and the object-oriented paradigm permits more flexibility when compared to a functional approach.

### Identification and categorization of behavioral events from raw Imetronic and other behavioral files

A necessary step to the combination of operant behavior and in vivo neuroimaging techniques is assuring that the events that are analyzed are categorically identical and can be pooled to uncover neurobiological signatures of the event of interest.

As there are many events occurring in self-administration including presentations of discriminative stimuli representing drug availability or unavailability, conditioned stimuli paired with reward delivery, reward delivery itself, drug seeking/taking behaviors, and more depending on the paradigm, a quick way to categorize events for subsequent analysis is highly beneficial.

Identification of identical behavioral events can also be incredibly beneficial in the scope of other behavioral tests. For example, through the use of the Behavior module, quick identification of regressive and perseverative errors in operant cognitive flexibility tasks can be produced by quick modification and customization of the configuration file. By extension, the tool can be used to analyze data from 5-choice serial reaction time tasks (5-CSRTTs) to tag the behavioral events associated with impulsive action, attention, and reaction times.

Regardless of the behavioral paradigm, ensuring that the timestamps used for subsequent neurobiological analysis are identical is necessary to uncover neurobiological correlates of complex behaviors. The Behavior module within *Pyfiber* helps do that.

### Signal processing for fiber photometry analysis

Regarding the signal processing techniques, we implemented the most commonly used techniques- ΔF/F and Z-score. Introduction of a new type of signal processing, if necessary, can be done due to the flexibility of the library.

Another clear benefit of *Pyfiber* in the perspective of signal processing is the flexibility that is permitted with peak analysis. With *Pyfiber*, similar to GuPPY, the rolling window for transient detection can be modified. However, *Pyfiber*, unlike GuPPY, permits more accessible modifications to the thresholds necessary to define transients.

Signal analysis can then be compared between groups to identify differences in neural activity. The type of signal processing techniques applied within *Pyfiber* was used to identify signatures of future stress susceptibility through the identification of differences in transient amplitude in medium spiny neurons in the nucleus accumbens^10^. Similar studies can be done using *Pyfiber*’s Fiber module to identify potential signatures of vulnerability to addiction, as well as other disorders.

### Selection of behavioral timestamps of interest and combination with photometry analysis

As previously discussed, the extraction and identification of identical behavioral events can be used to consequently analyze neural signatures surrounding different types of events within the same experiment. In addition, being able to easily select a specific operant response within the fixed ratio series (e.g. first/last nose-poke or lever press of the series…) can be meaningful to the study of reward seeking mechanisms. Thus, in a procedure aimed at testing motivation (i.e. a progressive ratio test) for a 10% sucrose solution in mice, the final nose-poke before the mice stopped working had the highest paranigral Ventral Tegmental Area response when compared to the other nose-pokes, uncovering subtle relationships between neuronal activity and behavioral responding in a motivational task^15^. In the same idea, we uncovered that different nose-pokes in the fixed ratio 5 (FR5) series have different neural signatures (*data not shown*).

In the scope of addiction, it can be meaningful to question addiction-related changes in neural signatures of drug consumption^11^, but also in neural signatures of environmental cues that have acquired drug-like effects through classical conditioning, which can promote relapse to drug use^16^. Studies using fiber photometry have explored neural correlates of variations in expression of cue-guided approach behavior^17^ or neural correlates of an exposure to a drug-associated context using conditioned place preference^18,19^ paving the way for the study of such responses in operant settings.

Dissection of neural correlates of other adaptive/maladaptive behaviors can be done through the combination of fiber photometry with operant tasks such as cognitive flexibility tasks or 5-CSRTT. The combination of complex cognitive testing and fiber photometry can contribute to the identification of the neural correlates involved in specific error types within cognitive flexibility paradigms as well as neural correlates related to impulsive action, attention, and reaction times through dissection of behavioral responses in 5-CSRTT trials^20^.

Additionally, the dissection of phasic and tonic neurotransmitter dynamics in response to drug delivery is also feasible through fiber photometry. However, it has yet to be applied to paradigms with contingent drug delivery^21^.

Being able to run refined analysis of complex decision-making processes in relation to fiber photometry signals will uncover important neural signatures in behaving animals with minimal manipulation of normal physiological states. These techniques can be especially useful when considering the pathological behavioral disruptions that occur in individuals with problematic substance use or other disorders that are currently modeled in rodents through complex behavioral paradigms. In addition, Pyfiber is particularly useful for a rapid assessment of the quality of effects. The ability to swiftly process individual files through the modules to evaluate the intensity of signals is highly valuable before conducting complex analysis on complete data sets.

### Limitations of Pyfiber

There are some limitations that must be acknowledged and addressed in future updates. One primary limitation is that the Behavior module can only directly process raw data from Imetronic software. Data from other behavioral systems need to be reformatted before being used in *Pyfiber*. Since many researchers utilize behavioral apparatus from other companies (e.g. Med Associates), it would be beneficial to expand *Pyfiber*’s capability to directly handle raw data from other behavioral systems.

Likewise, currently *Pyfiber* only supports fiber photometry files from Doric systems. As fiber photometry data files are relatively similar across most systems, the conversion from other systems to the Doric layout is quite simple. However, in a future version of *Pyfiber*, there will be the possibility to upload data types from other photometry systems. Lastly, another limitation is that currently, *Pyfiber* is only able to analyze single-site recordings. Given that researchers are now using multi-site recordings, it will be interesting to update Pyfiber to enable multi-site analysis.

In conclusion, *Pyfiber* can be a major asset to fluorescent imaging studies during operant behavior and favors their implementation, which remains limited. This open-source analytical python library helps eliminate analytical difficulties that behavioral labs face when implementing bulk fluorescent imaging techniques. Above all, *Pyfiber* permits a selective and exhaustive collection and analysis of the various events occurring during FP recordings to optimize data analysis. By bridging the analytical gap between behavioral events occurring in an operant behavior paradigm with fiber photometry signals, the use of *Pyfiber* and its adaptation to other set-ups open the door to an exhaustive analysis of the neuronal signature of the events occurring while freely behaving animals are tested in complex protocols.

*Pyfiber* is a project that aims to contribute by lightening the analytical burden underlying the combination of fluorescent imaging and behavioral neuroscience, contributing to advancement of understanding of neural dynamics during complex behaviors. The development of *Pyfiber* is a continuous project that will evolve and develop in function of advances in the field and feedback from users and potential users.

### Supplementary Information

Supplementary Legends.

Supplementary Information.

Supplementary Video 1.

Supplementary Video 2.

Supplementary Video 3.

## Data Availability

*Pyfiber* is developed for Python 3.10 and is compatible with Python 3.11. The library is available from the Python pip package manager by executing the following command in a terminal: ‘pip install pyfiber’. The source code, documentation which is automatically built with Sphinx, and issue tracker are also available from the following Github repository: https://gitlab.com/inserm-u1215/pyfiber. *Pyfiber* can be integrated into a homemade command-line application and interfaced in a notebook. For this, we recommend the Anaconda environment. Conditions of use and access are described step by step in the results section.
