# Supplementary material for: Integrating operant behavior and fiber photometry with the open-source python library Pyfiber
Source: Sci Rep. 2023 Oct 2;13:16562. doi: 10.1038/s41598-023-43565-1 (PMC10545777; doi:10.1038/s41598-023-43565-1)
Supplement: Supplementary file 1 — Supplementary Legends. [file 41598_2023_43565_MOESM1_ESM.pdf]

Legend to video 1: video 1 demonstrates a step-by-step installation process for Anaconda, Jupyter Notebook, and Pyfiber on your computer. You can find a repository containing useful links on how to install and use *Pyfiber*, along with example data, on GitLab at the following link: https://gitlab.com/inserm-u1215/pyfiber.

Legend to video 2: video 2 demonstrates how to perform analyses using *Pyfiber*. It provides step-by-step instructions for two prototypical types of analyses: the first type focuses on experimenter-scheduled events, while the second type covers the analysis of non-scheduled events resulting from animal behavior.

Legend to video 3: video 3 demonstrates how to modify the configuration file if necessary (refer to additional details in the SI).
