# Supplementary material for: Integrating operant behavior and fiber photometry with the open-source python library Pyfiber
Source: Sci Rep. 2023 Oct 2;13:16562. doi: 10.1038/s41598-023-43565-1 (PMC10545777; doi:10.1038/s41598-023-43565-1)
Supplement: Supplementary file 2 — Supplementary Information. [file 41598_2023_43565_MOESM2_ESM.docx]

**Supplementary Information**

Integrating Operant Behavior and Fiber Photometry with the Open-Source Python Library Pyfiber

**Dana Conlisk^#1^, Matias Ceau^#1^, Jean-François Fiancette^1^, Nanci Winke^1^, Elise Darmagnac^1^, Cyril Herry^1^, Véronique Deroche-Gamonet^1^**

^1^ University of Bordeaux, INSERM, Neurocentre Magendie, U1215, F-33000 Bordeaux, France

# Contributed Equally

*Correspondence to: [veronique.deroche@inserm.fr](mailto:veronique.deroche@inserm.fr)

**Supplementary Information**

**1. Supplemental Methods**

**1.1. Subjects**

Male Sprague Dawley CD rats (experiment 1 - cocaine intravenous self-administration: n=14; experiment 2 - sucrose oral self-administration: n=5) weighing 250-300g at their arrival were single housed in a temperature (22 ± 1°C) and humidity (60 ± 5%) controlled vivarium on a reverse-light cycle (ON 19h00, OFF 7h00). Food and water were provided *ad libitum*. Rats were handled daily before experimentation began.

**1.2. Surgery**

*Stereotaxic:* After 3-6 days of habituation, the animals were anesthetized with a gas containing isoflurane (Vetflurane, Virbac, France). Ambient compressed air was used as the carrier for isoflurane. Anesthesia was induced with a 5% isoflurane concentration and maintained with a 2% isoflurane concentration. Anesthetized rats were placed in a stereotaxic frame (Kopf). During surgery, body temperature was monitored and maintained at 37 degrees Celsius. In experiment 1, GCaMP6f (Addgene, pENN.AAV.CamKII.GCaMP6f.WPRE.SV40- original titer: 2.30E+13 vg/ml, final titer: 2.30E+12 vg/ml) was used as the viral vector while in experiment 2, dLight1.3b (Zurich Viral Vector Core, ssAAV-9/2-hSyn1-chl-dLight1.3b-WPRE-bGHp(A)- original and final titer: 7.9E12 vg/ml) was used. Using a glass micropipette, 500 nL of the virus was injected into the prelimbic cortex (Experiment 1: AP: +3.0, ML: - 0.6, DV: -3.5 from skull) or the nucleus accumbens core (Experiment 2: AP: 1.3, ML: -1.55, DV: -7.0) at a rate of approximately 250 nanoliters/minute. The micropipette was allowed to stay in place for 10 minutes to allow for diffusion. A hollow blunted tip 27G needle (outer diameter 410 micrometers) was lowered to the placement of the fiber then removed to prevent tissue accumulation under the recording area of the fiber. Lastly, the fiber (400 μm core, 0.66 NA, 5 mm in length for prelimbic cortex and 8.5 mm for nucleus accumbens core; Doric Lenses) was inserted and secured with an opaque dental cement added with carbon powder to block any light from passing through (C&B Metabond, Parkell). The analgesic metacam (1 mg/kg, SC) was administered 10 to 15 minutes pre-operatively. Additionally, lidocaine was locally injected at the incision location.

*Intravenous catheterization:* Four weeks after the stereotaxic surgery, the rats underwent intravenous catheterization. A silastic catheter (internal diameter = 0.28 mm; external diameter = 0.61 mm; dead volume = 12 µl) was implanted in the right jugular vein under isoflurane anesthesia. The proximal end of the catheter was inserted into the right atrium, passed under the skin, and the base emerged from the mid scapular region. Rats were treated post-op with metacam (1 mg/kg, SC) and allowed to recover for 5 to 9 days after surgery before self-administration began.

**1.3. Histology**

At the end of the experiment, the rats were deeply anesthetized with an intraperitoneal injection of a mix of pentobarbital and lidocaine (200 mg/kg pentobarbital / 20 mg/kg lidocaine). The animals were transcardially perfused with 4% paraformaldehyde (PFA) and then their brains were removed and stored in 4% PFA until they were sliced. The brains were sliced (slice thickness 50 μm) and immediately placed on glass slides. Proper fiber placement and adequate virus expression was analyzed using a Nikon Eclipse inverted microscope (Ti-U) **(Fig. S1)**.

**1.4. Self-administration apparatus**

The self-administration setups were composed of plexiglass and metal (Imetronic, Marcheprime, France). Each chamber (40 cm long x 30 cm wide x 52 cm high) was encased within a larger opaque box equipped with exhaust fans that assured air circulation and masked background noise. The configuration of the boxes varied according to the type of experiment (cocaine intravenous or sucrose oral self-administration). In experiment 1 (cocaine self-administration), animals were placed in a chamber where their chronically implanted intra-cardiac infusion catheter was connected to a pump-driven syringe. Two holes, located on opposite sides of the chamber at 5 cm from the grid floor, were used to record nose-poking. The chambers were equipped with a blue cue light (1.8 cm in diameter) located on the opposite wall from the active hole at 33 cm from the grid, a white house light at the top of the chamber, and a white cue light (1.8 cm in diameter- producing 5 Lux) located 9.5 cm above the active hole. During the sessions in experiment 2 (sucrose oral self-administration), animals were simply placed in an operant box at the beginning of the session. Two holes, located on the same side of the chamber at 5 cm from the grid floor, were used to record nose-poking. A cup or licking device (licker) was located on the same side and between the two holes.

The chambers were equipped with a blue cue light (1.8 cm in diameter) located above the active hole at 33 cm from the grid, a white house light at the top of the chamber, and a white cue light (1.8 cm in diameter- producing 5 Lux) located 9.5 cm above the active hole. Experimental contingencies were controlled and data collected with a PC-windows-compatible software (Imetronic, Marcheprime, France).

**1.5. Self-administration training**

**1.5.1 Intravenous cocaine self-administration**

*Basal self-administration session*. All experiments were performed in the dark phase of the light/dark cycle. The daily sessions consisted of three drug periods (40 min) separated by two no drug periods (15 min). Drug periods were characterized by illumination of the chamber by a blue light (LED2) while no drug periods were characterized by illumination of the chamber by a white house light (HLED) **(Fig. 1C)**. Inactive nosepokes during the duration of the sessions were recorded but had no scheduled consequences and active nosepokes during the no drug period were recorded but had no scheduled consequences. Animals were trained on a fixed ratio 3 (FR3) of responding for the first three days of self-administration (max infusions 25, 25, 30, respectively), then continued on an FR5 ratio of responding for the duration of the experiment (max infusions 30, then 35). Once the animals reached the necessary number of nosepokes for infusion, the white cue light (LED1) illuminated for four seconds, with the pump being activated (delivering 46 microliters, 0.8 mg/kg/inf) one second after the illumination of the white light. Then, both the white cue light and blue light were turned off during the 40 second time-out period **(Fig. 1B)**. Active and inactive responding during the time-out period were recorded but had no scheduled consequences.

**1.5.2 Sucrose self-administration**

*Basal self-administration session*. All experiments were performed in the dark phase of the light/dark cycle. The daily sessions consisted of a 90-min sucrose access period. It was characterized by illumination of the chamber by a blue light (LED2) **(Fig. 1F)**. Inactive nosepokes during the duration of the sessions were recorded but had no scheduled consequences. Animals were trained on a fixed ratio 1 (FR1) of responding for the first three days of self-administration, then continued on an FR3 ratio of responding for the duration of the experiment. Once the animals reached the necessary number of nosepokes for sucrose delivery, the white cue light (LED1) illuminated for four seconds, with the pump being activated one second after the illumination of the white light. Then, both the white cue light and blue light were turned off during the 40 second time-out period **(Fig. 1E)**. Active and inactive responding during the time-out period were recorded but had no scheduled consequences.

**1.6. Fiber photometry system**

Fiber photometry recordings were done with a 1-site Doric Lenses photometry system (Doric Lenses, Quebec, Canada). The intensity of the 405 and 465 nm lights was sinusoidally modulated at 572 and 268 Hz, respectively. Light was coupled to a filter cube (FMC4, Doric Lenses), converging onto a patch cord that was connected to the animal’s implanted optical fiber. Fluorescence collection was through the same patch cord, then passed to a photoreceiver (Visible Femtowatt Photoreceiver Module Model 2151, Newport). Output control and data acquisition were synchronized through a Doric Lenses Photometry Console, then passed to a PC that ran Doric Neuroscience Studio software. Data was low pass filtered with a cutoff frequency of 12 Hz.

**1.7. Fiber recording during self-administration**

The fiber photometry self-administration sessions were identical to the basal self-administration sessions, except that the rats were connected to a dual pharmacology/optic fiber-motorized commutator (Imetronic, Marcheprime, France) which ensured that the patch cord and the line containing the cocaine solution rotated smoothly and in parallel to prevent tangling of the two lines. A TTL output connected the Imetronic behavioral system to the Doric Lenses fiber photometry system.This TTL output initiated and stopped the fiber photometry recordings.

Fiber recordings may take place at any moment throughout the operant protocol as they are initiated and stopped by the TTL output from the Imetronic system. The duration of the recordings in our experimental protocols ranged from 60 sec to 16 minutes, depending on our experimental questioning. We provide examples of cocaine self-administration fiber recordings that were taken during various phases of the self-administration session. These phases include the initiation of the first drug period (D1), the transition from D1 to the first no drug period (ND1), the period from the end of ND1 to the beginning of the second drug period (D2), during D2, and the transition from D2 to the second no drug period (ND2). Additionally, we have recordings that span from the conclusion of the first drug period (D1), encompassing the entire no drug period (ND1) and concluding after the transition to the second drug period (D2), as indicated by the green boxes in **Fig. 1C**. For sucrose self-administration sessions, we conducted a single fiber photometry recording at the start of the self-administration session, represented by the green box in **Fig. 1F**.

**2. Supplemental Results**

**2.1. How to install and use *Pyfiber***

All the steps described below are detailed in **Video 1**.

Where to find the *Pyfiber* Library (*Python Package Index- PyPi):* <https://pypi.org/project/pyfiber/>

Help on how to use *Pyfiber* (*Read The Docs):*<https://pyfiber.readthedocs.io/en/latest/>

Repository where to find the links cited above and example data/Jupyter notebooks for using *Pyfiber (Gitlab):*<https://gitlab.com/inserm-u1215/pyfiber>

*Pyfiber* can be integrated into a homemade command-line application and interfaced in a notebook. For this, we recommend the Anaconda environment.

***Installation of Anaconda, Jupyter Notebook, and Pyfiber***

*Pyfiber* is recommended to be used through Jupyter Notebook. Guidance on how to install Anaconda and Jupyter notebook can be found here: [*https://sparkbyexamples.com/python/install-anaconda-jupyter-notebook/*](https://sparkbyexamples.com/python/install-anaconda-jupyter-notebook/)

Installation of *Pyfiber* can be done by typing “pip install pyfiber” in the Anaconda Prompt.

***Importing Pyfiber to Jupyter Notebook***

Before using any of the modules below, the user needs to import *Pyfiber* to the notebook. This can be done by calling the below command:

This command is done in all example notebooks provided on Gitlab. When executed, it shows the location of the configuration file.

**2.2. Description of the configuration file**

Lines 1-9 of the configuration file contain general information regarding the identification tags and nomenclature.

Line 4-5 indicates the automatic naming that was designed by our lab. It specifies that the folder containing the data files is formatted to contain information about the experiment, rat number, experiment type, and self-administration session. For example, in an example folder (AS21R_rat_12_SA7_j40), the experiment (AS21R), rat (12), experiment type (SA7), and session number (40) can automatically be extracted by *Pyfiber*.

The default behavior file type (line 12) is Imetronic. The default configuration of the behavior_time_ratio (line 13) is 1000, due to the behavior file containing information in milliseconds.

Lines 19-32 contain the definition of events that can be extracted from the raw Imetronic file and called for subsequent analysis.

Along the same lines, there are intervals that are calculated from the aforementioned events that can be used as inclusion or exclusion criteria for analysis: for example, taking nose-pokes only when HLED is on.

If there are other events or intervals of interest in analysis, such as levers or lickers, events can be added using the nomenclature found in rows 147-160. They just need to be added to the imetronic_events section.

For example, if levers are used instead of nose-pokes, there needs to be this addition to the imetronic_events section that contains the following imetronic configuration:

It is ‘conditional’ since the determination of the timestamp of this event is conditional on a 1 occurring in the ‘_V’ column in the Imetronic file when the [F, ID] is equal to [2,1]. This information can be found in the Imetronic manual.

Additionally, within our protocol, there are complexities that require us to add additional custom events and intervals that can be found in lines 63-103. These include drug periods (LED2 ON), no drug periods (HLED ON), time-out periods [LED1 ON (cocaine conditioned stimulus), and the subsequent dark period] (**Figure 1C**).

When dealing with data from non-Imetronic systems, the customization of the .yaml configuration file is only necessary within the 'basic_intervals' and 'custom' sections. Event names are derived from the column headers present in the imported data file. For example, if the imported data file contains column headers such as 'Dim1_on', 'Dim1_off', 'Dim2', and so on, the 'basic_intervals' section should be configured as demonstrated below:


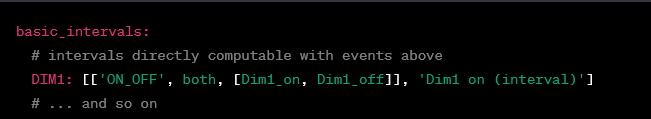


It is crucial to emphasize that, regardless of the specific system in use, events and intervals of interest must be explicitly defined in the .yaml configuration file in line with with the experiment's specific objectives.

In rows 106-126, there are the default plotting and color settings for events/intervals.

Next, the configuration file contains the default settings for the fiber photometry analyses. Changes in default settings can be done by changing this file and saving it. The next time the user imports *Pyfiber* to initialize analysis, the changed default settings will be taken into account. For example, if perievent analysis is always done on 5 seconds pre-event and 5 seconds post-event, change the white text in line 134 from [1.0, 1.0] to [5.0, 5.0].

Lastly, the configuration file contains the nomenclature from the Doric Lenses system that is used to define the isosbestic, calcium dependent, time, and TTL channels, as well as other nomenclature that is used to define the columns containing time, signal, or TTL data. These can be found at the top columns of the .csv files generated by the Doric system.

After confirming that the default settings are as desired, the modules can be called.

**2.3. Supplementary information on application of the component modules of *Pyfiber*: Fiber, Behavior (MultiBehavior and Behavior), and Session modules *Pyfiber* modules**

**2.3.1. Fiber Module**

An example Jupyter Notebook that can be used as a reference for the benefits of the Fiber module can be found and downloaded with the example file at:

**https://gitlab.com/inserm-u1215/pyfiber/-/tree/main/notebooks/Fiber**

**2.3.2. Behavior Module**

***Behavior***

An example Jupyter Notebook that can be used as a reference for the benefits of the behavior module can be found and downloaded with the example file at:

**https://gitlab.com/inserm-u1215/pyfiber/-/tree/main/notebooks/Behavior**

***MultiBehavior***

An example Jupyter Notebook that can be used as a reference for the benefits of the MultiBehavior module can be found and downloaded with the example files at:

**https://gitlab.com/inserm-u1215/pyfiber/-/tree/main/notebooks/MultiBehavior**

**2.3.3. Session**

Below is a tutorial explaining how to use the Session module to analyze a single recording session.

The Session module is used to analyze one behavioral and fiber data file in parallel. It uses the **Behavior**, **Fiber**, and **Analyze** modules.

The corresponding Jupyter Notebook can be found and downloaded with the example file at:

**https://gitlab.com/inserm-u1215/pyfiber/-/tree/main/notebooks/Session**

***Analysis of fiber photometry signals in response to the first drug to no drug shift in a single self-administration session***

**Step 1 *(Session Jupyter Notebook: #1)*:** Import *Pyfiber* (see **Results paragraph 3**).

**Step 2 *(Session Jupyter Notebook: #2)*:** Create a session object by calling pf.Session() and indicating the file path to the fiber and behavioral files in the parenthesis.

When working with systems other than Imetronic, a second parameter should be provided as the 'filetype' parameter:

sess=pf.Session(fiber='C:/__FILEPATH__.csv', behavior='C:/__FILEPATH__.dat', filetype='Other_system')

**Step 3 *(Session Jupyter Notebook: #3)*:** Identify timestamps of the events of interest. Shown here is extraction of both the timestamps of injections in the first drug period and the timestamps of the switch from the first drug to the no drug period.

**Step 4 *(Session Jupyter Notebook: #6)*:** Using the timestamp of the switch, perievent analysis can be done by calling the Analyze module.

**Step 5 *(Session Jupyter Notebook: #7)*:** Like in the MultiSession module, the signal can be plotted by calling .plot().

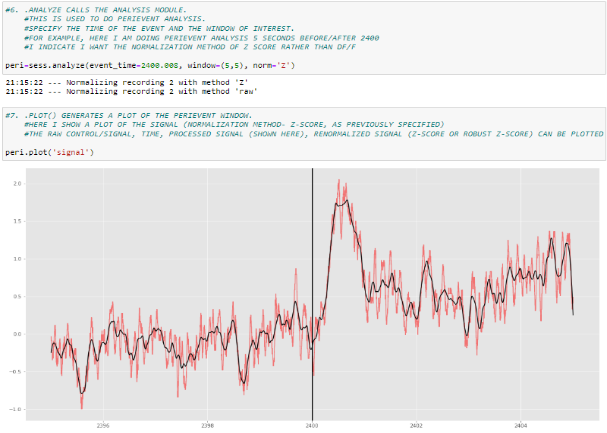


**Step 6 *(Session Jupyter Notebook: #14)*:** If the interest is comparing the peak frequency before and after the event occurs, this can be done by calling the analysis of interest within the perievent object.

**2.4. Features and Commands associated with the different modules**

**2.4.1. MultiSession Module**

Below is a table containing a selection of the most pertinent features and the commands associated with the MultiSession module:

multisessionx = pyfiber.MultiSession('path_to_the_folder_with_subfolders_containing_a _behavior_and_fiber_file')

When working with systems other than Imetronic, a second parameter should be provided as the 'filetype' parameter:

multisessionx = pyfiber.MultiSession('path_to_the_folder_with_subfolders_containing_a _behavior_and_fiber_file', filetype=’Other_system’)

| **Method** | **Feature** | **Command example** | **Jupyter Notebook Title** |
| --- | --- | --- | --- |
| Details | Shows a list containing the experiment, rat number, experiment type, session number, ID, and session tag | multisessionx.details | Multisession #3 |
| Folder | Shows the location of the folder that contains the subfolders containing the data files | multisessionx.folder | Multisession #4 |
| Names | Shows the session tags | multisessionx.names | Multisession #5 |
| Removed | Shows removed sessions (due to us having a problem with the Imetronic system- theoretically no one else will have this problem) | multisessionx.removed |  |
| Sessions | A dictionary containing the session tag and the locations for the behavior and fiber data files | multisessionx.sessions | Multisession #6 |
| Compare Behavior | Output is a heat map and a plot containing a visual representation of the differences in behavior (a single event). Used to visualize behavioral differences between sessions/rats. | multisessionx.compare_behavior(‘np1’) | Multisession #7 |

| **Analyze** | | | |
| --- | --- | --- | --- |
| Analyze | Prepare analyzed fiber data related to defined perievent window. The analysis needs to be put in a variable which is used to get graphs or dataframes. | MultiAnalyze = multisession.analyze('switch_1_D', window=(10,10)) | Multisession #8, #16 |
| Plot | Plot the perievent analysis (either time, raw control, raw signal, processed signal, z-score, or robust z-score). | MultiAnalyze.plot(‘time’)  MultiAnalyze.plot(‘raw_control’)  MultiAnalyze.plot(‘raw_signal’)  MultiAnalyze.plot(‘signal’)  MultiAnalyze.plot(‘zscores’)  MultiAnalyze.plot(‘rob_zscores’) | Multisession #9, #17 |
| **Data** | **Return a dataframe with each event as a row and the columns containing all information below in *italic*, which can also be called individually** | **MultiAnalyze.data** | **Multisession #10, #18** |
| *Verification of filepath* | *The filepath for the behavioral or fiber data files*  Can also be called without .data | MultiAnalyze.fiberfile  MultiAnalyze.behaviorfile | Multisession #11 |
| *Normalization method* | *Verification of the normalization method used (should be what is indicated in the configuration file unless otherwise indicated* | MultiAnalyze.normalisation | Multisession #11 |
| *Verification of event time* | *Output is the time of the event* | MultiAnalyze.event_time | Multisession #11 |
| *Verification of perievent window* | *Output is the perievent window* | MultiAnalyze.window | Multisession #11 |
| *Sampling rate of recordings used* | *Output is the sample rate of the recording used* | MultiAnalyze.sampling_rate | Multisession #11 |
| *Recording number of recording used* | *Output is the recording number of the recording used for the perievent analysis* | MultiAnalyze.data.rec_number | Multisession #11 |
| *Pre/post peak frequency* | *Output is the frequency of peaks/second either pre or post event* | MultiAnalyze.pre_peak_frequency  MultiAnalyze.data.post_peak_frequency | Multisession #11 |
| *Average pre/post peak amplitude* | *Output is the average amplitude of the peaks either pre or post event (either ΔF/F or Z-score)* | MultiAnalyze.pre_peak_AVG_dFF  MultiAnalyze.post_peak_AVG_dFF  MultiAnalyze.pre_peak_AVG_Z  MultiAnalyze.post_peak_AVG_Z | Multisession #11 |
| *Maximum pre/post peak amplitude* | *Output is the maximum peak amplitude either pre or post event (either ΔF/F or Z-score)* | MultiAnalyze.pre_peak_max_ dFF  MultiAnalyze.post_peak_max_dFF  MultiAnalyze.pre_peak_max_Z  MultiAnalyze.post_peak_max_Z | Multisession #11 |
| *Pre/post AUC* | *Output of the area under the curve of the pre/post signal of choice (raw (calcium dependent channel), processed signal, robust Z-score, or Z-score)* | MultiAnalyze.pre_raw_AUC  MultiAnalyze.post_raw_AUC  MultiAnalyze.preAUC  MultiAnalyze.postAUC  MultiAnalyze.preRZ_AUC  MultiAnalyze.postRZ_AUC  MultiAnalyze.preZ_AUC  MultiAnalyze.postZ_AUC | Multisession #11 |
| *Pre/post average values* | *Output is the average value of the pre/post signal of choice (raw(preprocessed), robust Z-score, or Z-score)* | MultiAnalyze.preAVG_dF  MultiAnalyze.postAVG_dF  MultiAnalyze.preAVG_RZ  MultiAnalyze.postAVG_RZ  MultiAnalyze.preAVG_Z  MultiAnalyze.postAVG_Z | Multisession #11 |
| Full Data | Return a dataframe with each event as a row and the columns containing all information above in *italic* and the dataframes below in **bold** (which can also be called individually by calling MultiAnalyze.full_data._____) | MultiAnalyze.full_data | Multisession #12 |
| **Pre/post**  **event**  **dataframes** | **Output of a dataframe containing the time, raw (control or signal), processed data, or re-normalized data (Robust Z-scores or Z-scores) selectively either before or after the event occurs** | MultiAnalyze.pre_time  MultiAnalyze.post_time  MultiAnalyze.pre_raw_ctrl  MultiAnalyze.post_ raw_ctrl  MultiAnalyze.pre_raw_sig  MultiAnalyze.post_ raw_sig  MultiAnalyze.preevent  MultiAnalyze.postevent  MultiAnalyze.pre_Rzscores  MultiAnalyze.post_Rzscores  MultiAnalyze.pre_zscores  MultiAnalyze.post_zscores | Multisession #13 |
| **Raw Data** | **An array containing the time and raw data (raw signal and control channels) or the time and processed signal for the entire recording that was used for the perievent analysis** | MultiAnalyze.rawdata  MultiAnalyze.recordingdata | Multisession #14 |
| **Export individual signals** | **An array containing only the time, raw control channel, calcium dependent channel, processed data, or re-normalized data (Robust Z-scores or Z-scores) selectively during the perievent time period** | MultiAnalyze.time  MultiAnalyze.raw_control  MultiAnalyze.raw_signal  MultiAnalyze.signal  MultiAnalyze.rob_zscores  MultiAnalyze.zscores | Multisession #13 |
| **Data** | **Perievent data (time and processed signal (ΔF/F or Z-score)** | **MultiAnalyze.full_data.data** | Multisession #13 |
| Export average signals | An array containing the mean values of all recordings | MultiAnalyze.EPOCH  MultiAnalyze.RAW_CONTROL  MultiAnalyze.RAW_SIGNAL  MultiAnalyze.ROB_ZSCORES  MultiAnalyze.SIGNAL  MultiAnalyze.TIME  MultiAnalyze.WINDOW  MultiAnalyze.ZSCORES |  |
| Export interpolated signals | An array with interpolated signals (due to slight differences that exist between different recordings) | MultiAnalyze.interpolated_epoch  MultiAnalyze.interpolated_raw_control  MultiAnalyze.interpolated_raw_signal  MultiAnalyze.interpolated_rob_zscores  MultiAnalyze.interpolated_signal  MultiAnalyze.interpolated_time  MultiAnalyze.interpolated_zscores | Multisession #15 |

**2.4.2. Session Module**

Below is a table containing a selection of the most pertinent features and the commands associated with the Session module:

session = pf.Session('path_to_the_folder_with_behavior_and_fiber_file')

When working with systems other than Imetronic, a second parameter should be provided as the 'filetype' parameter:

session = pf.Session('path_to_the_folder_with_behavior_and_fiber_file', filetype=’Other_system’)

| **Method** | **Feature** | **Command example** | **Jupyter Notebook Title** |
| --- | --- | --- | --- |
| **Behavior** | | | |
| All Behavior Methods (see table 1) | All features available in pyfiber.Behavior | session.behavior.______ | Session #3, #4 |
| **Fiber** | | | |
| All Fiber Methods (see table 3) | All features available in pyfiber.Fiber | session.fiber.______ | Session #5 |
| **Analyze** | | | |
| Analyze | Prepare analyzed fiber photometry data related to defined event extracted from the perievent window. Need to put it in a variable and then use it to get graphs or dataframes. | objAnalyze = session.analyze('np1', window=(0.5, 0.5)) | Session #6 |
| Plot | Plot the perievent analysis (either time, raw control, raw signal, processed signal, z-score, or robust z-score) | objAnalyze.plot(‘time’)  objAnalyze.plot(‘raw_control’)  objAnalyze.plot(‘raw_signal’)  objAnalyze.plot(‘signal’)  objAnalyze.plot(‘zscores’)  objAnalyze.plot(‘rob_zscores’) | Session #7 |
| Smooth | Outputs a smoothed array (using the Savitzky-Golay filter) | objAnalyze.smooth(‘time’)  objAnalyze. smooth (‘raw_control’)  objAnalyze. smooth (‘raw_signal’)  objAnalyze. smooth (‘signal’)  objAnalyze. smooth (‘zscores’)  objAnalyze. smooth (‘rob_zscores’) | Session #8 |
| Data | Perievent data (time and processed signal (dF/F or Z-score) | objAnalyze.data | Session #9 |
| Full Data Frame- Raw and/or processed | An array containing the time and raw data (raw signal and control channels) or the time and processed signal for the entire recording that was used for the perievent analysis | objAnalyze.rawdata  objAnalyze.recordingdata | Session #10 |
| Export signal | An array containing only the time, raw control channel, calcium dependent channel, processed data, or re-normalized data (Robust Z-scores or Z-scores) selectively during the perievent time period | objAnalyze.time  objAnalyze.raw_control  objAnalyze.raw_signal  objAnalyze.signal  objAnalyze.rob_zscores  objAnalyze.zscores | Session #11 |
| Pre/post  event  dataframes | Output of a dataframe containing the time, raw (control or signal), processed data, or re-normalized data (Robust Z-scores or Z-scores) selectively either before or after the event occurs | objAnalyze.pre_time  objAnalyze.post_time  objAnalyze.pre_raw_ctrl  objAnalyze.post_ raw_ctrl  objAnalyze.pre_raw_sig  objAnalyze.post_ raw_sig  objAnalyze.preevent  objAnalyze.postevent  objAnalyze.pre_Rzscores  objAnalyze.post_Rzscores  objAnalyze.pre_zscores  objAnalyze.post_zscores | Session #12 |
| Pre/post AUC | Output of the area under the curve of the pre/post signal of choice (raw (calcium dependent channel), processed signal, robust Z-score, or Z-score) | objAnalyze.pre_raw_AUC objAnalyze.post_raw_AUC  objAnalyze.preAUC  objAnalyze.postAUC  objAnalyze.preRZ_AUC  objAnalyze.postRZ_AUC  objAnalyze.preZ_AUC  objAnalyze.postZ_AUC | Session #13 |
| Pre/post average values | Output is the average value of the pre/post signal of choice (raw(preprocessed), robust Z-score, or Z-score) | objAnalyze.preAVG_dF  objAnalyze.postAVG_dF  objAnalyze.preAVG_RZ  objAnalyze.postAVG_RZ  objAnalyze.preAVG_Z  objAnalyze.postAVG_Z | Session #13 |
| Pre/post peak frequency | Output is the frequency of peaks/second either pre or post event | objAnalyze.pre_peak_frequency  objAnalyze.post_peak_frequency | Session #14 |
| Average pre/post peak amplitude | Output is the average amplitude of the peaks either pre or post event (either ΔF/F or Z-score) | objAnalyze.pre_peak_avg_dFF  objAnalyze.post_peak_avg_dFF  objAnalyze.pre_peak_avg_Z  objAnalyze.post_peak_avg_Z | Session #14 |
| Maximum pre/post peak amplitude | Output is the maximum peak amplitude either pre or post event (either ΔF/F or Z-score) | objAnalyze.pre_peak_max_dFF  objAnalyze.post_peak_max_dFF  objAnalyze.pre_peak_max_Z  objAnalyze.post_peak_max_Z | Session #14 |
| Verification of filepath | The filepath for the behavioral or fiber data files | objAnalyze.fiberfile  objAnalyze.behaviorfile | Session #15 |
| Normalization method | Verification of the normalization method used (should be what is indicated in the configuration file unless otherwise indicated | objAnalyze.normalisation | Session #16 |
| Verification of event time | Output is the time of the event | objAnalyze.event_time | Session #17 |
| Verification of perievent window | Output is the perievent window | objAnalyze.window | Session #17 |
| Sampling rate of recordings used | Output is the sample rate of the recording used | objAnalyze.sampling_rate | Session #18 |
| Recording number of recording used | Output is the recording number of the recording used for the perievent analysis | objAnalyze.rec_number | Session #18 |

**2.4.3. Fiber Module**

Below is a table containing a selection of the most pertinent features and the commands associated with the Fiber module:

fiber = pf.Fiber('path_to_the_fiber_file')

| **Method** | **Feature** | **Command example** | **Jupyter Notebook Title** |
| --- | --- | --- | --- |
| Plot | Show graphs with Fiber data. | fiber.plot() | Fiber #3 |
| Plot transients | Show a graph with transients among time. | fiber.plot_transients() | Fiber #4 |
| Export to CSV | Export data to a CSV file. This reformats the fiber data to the configuration that is used in GuPPY. | fiber.to_csv() | Fiber #5 |
| Normalize | Normalize data with specified method. Can be with either ΔF/F (‘F’) or Z-scores (‘Z’). Can be specific recordings, but the default is all. | fiber.norm(method='F', rec=1) | Fiber #6, #7 |
| Peaks | Return a dictionary containing a data frame with peak data (timestamps and amplitude (ΔF/F and Z-score)) for all recordings. | fiber.peaks | Fiber #8 |
| PeakFA | After indicating the boundaries, it returns the peak frequency, average, and maximum amplitude of peaks (both ΔF/F and Z-score) | fiber.peakFA(2400, 2405) | Fiber #9 |
| Get | Extracts data array for a specific column of a recording. Can be either ‘time,’ ‘signal,’ or ‘control.’ | fiber.get('signal') | Fiber #10 |

**2.4.4. Behavior Module**

***Behavior***

Below is a table containing a selection of the most pertinent features and the commands associated with the Behavior module:

behavior = pf.Behavior('path_to_the_behavior_file')

When working with other systems than Imetronic, a second parameter should be passed:

behavior = pf.Behavior('path_to_the_behavior_file', ‘Other_system’)

| **Method** | **Feature** | **Command example** | **Jupyter Notebook Title** |
| --- | --- | --- | --- |
| Summary | Show a graphical overview of main events and intervals (as defined in the configuration file). | behavior.summary() | Behavior #5 |
| Figure | Plot event of interest along time. | behavior.figure(‘inj1’) | Behavior #6 |
| Total | View count of all events | behavior.total | Behavior #7 |
| Raw | View raw data | behavior.raw | Behavior #8 |
| Data | Output is an array containing all events and when they occur (in which intervals) | behavior.data | Behavior #9 |
| Timestamps | Return array with timestamps of an event according to filters. | behavior.timestamps('inj1', interval = ('D_1')) | Behavior #10, #11 |
| Export Timestamps | Same as Timestamps with a graphical representation of events and inclusion/exclusion criteria. | behavior.export_timestamps('inj1', interval = ('D_1')) | Behavior #12 |
| Events | If nothing is in the (), there will be an output containing all the events of the session.  If an event is specified (e.g, ‘inj1’), the output will be all events that occur during fiber recording (when TTL1=ON). | behavior.event()  behavior.event(‘inj1’) | Behavior #13 |
| Intervals | Return list of all intervals defined in the configuration file. | behavior.intervals() | Behavior #14 |
| Specific Data visualization | Return a dataframe summarizing the Imetronic data from the dat file for a specific event. | behavior.get(‘INJ1’) | Behavior #15 |
| Movement | Produces a heatmap of localization in the box (in our case, left or right as we only have two beams). | behavior.movement() | Behavior #16 |

***MultiBehavior***

Below is a table containing a selection of the most pertinent features and the commands associated with the MultiBehavior module:

multibehavior = pf.MultiBehavior('path_to_the_folder_with_behavior_files')

When working with other systems than Imetronic, a second parameter should be passed:

multibehavior = pf.MultiBehavior('path_to_the_folder_with_behavior_files', ‘Other_system’)

| **Method** | **Feature** | **Command example** | **Jupyter Notebook Title** |
| --- | --- | --- | --- |
| Count | Returns a dataframe with the cumulative number of events per second. | multibehavior.count(‘np1’) | MultiBehavior #3 |
| Cumul | Show a graph with events accumulated over time animal per animal. | multibehavior.cumul(‘np1’) | MultiBehavior #4 |
| Show Rate | Show rate for all sessions for any given event (default window is 120s). Also, shows a graph including the 15^th^, 50^th^, and 85^th^ percentiles. | multibehavior.show_rate(‘np1’) | MultiBehavior #5 |
| Summary | Same as summary of Behavior module but for all the analyzed sessions. | multibehavior.summary() | MultiBehavior #6 |
| Timestamps of events | Shows a dataframe with each row as a .dat file and the columns containing the timestamps of the event or interval of interest. | multibehavior.inj1 multibehavior.led1_on  (and all other events or intervals within the configuration file) | MultiBehavior #7 |

**3. Supplemental Figure**

**
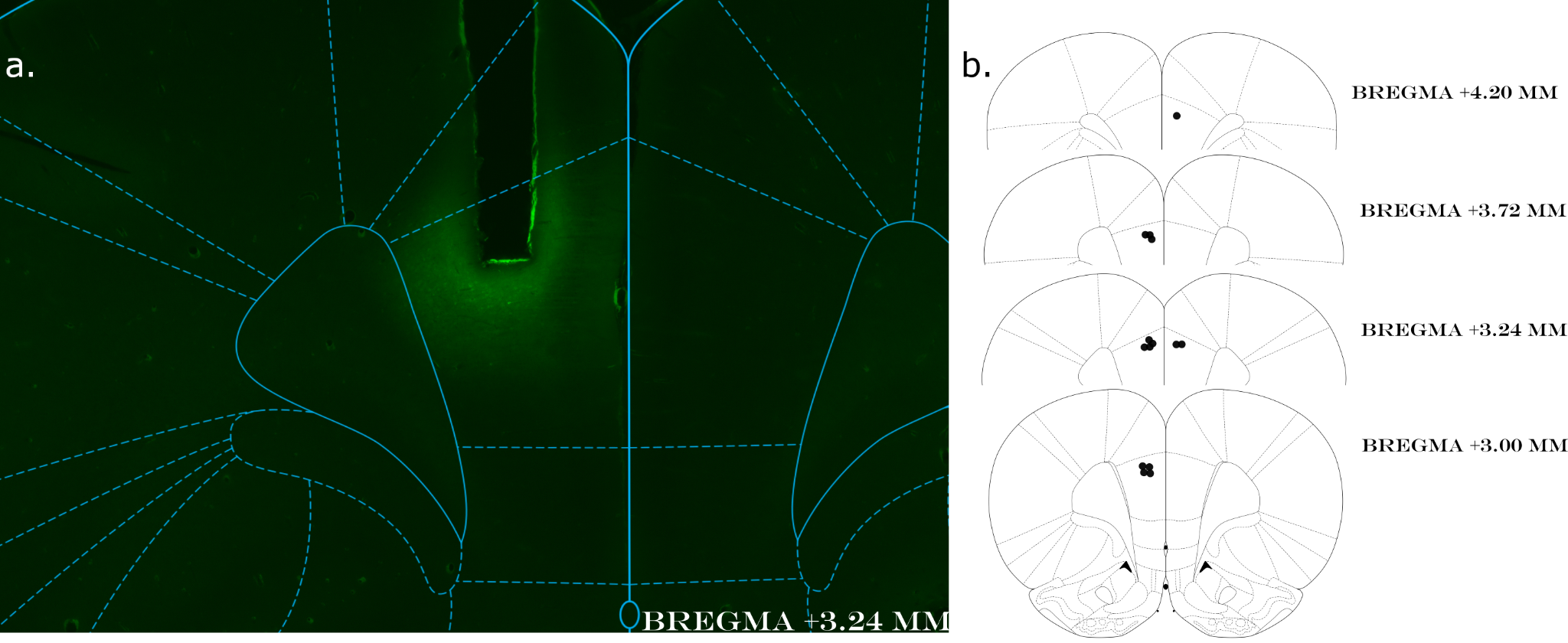
**

**Fig. S1:** **A.** Example of expression of the GCaMP6f in the Prelimbic Cortex (PL) attested by the green fluorescence emitted by the circularly permuted green fluorescent protein (cpGFP), and location of the tip of the optical fiber. B. Placements of the optical fiber in the 14 tested rats targeting the PL from AP +4.2 to AP +3 mm.
